# Low CRIM1 Levels Predict Poor Prognosis in Breast Cancer Patients

**DOI:** 10.3389/fonc.2022.882328

**Published:** 2022-05-06

**Authors:** Wei Wen, Baohong Jiang, Xi Cao, Liming Xie, Xiaoli Zhang, Yuehua Li, Rongfang He

**Affiliations:** ^1^ Department of Pathology, The First Affiliated Hospital, Hengyang Medical School, University of South China, Hengyang, China; ^2^ Department of Pharmacy, The First Affiliated Hospital, Hengyang Medical School, University of South China, Hengyang, China; ^3^ Department of Medical Oncology, The First Affiliated Hospital, Hengyang Medical School, University of South China, Hengyang, China

**Keywords:** breast cancer, CRIM1, poor prognosis, bioinformatics analysis, biomarker

## Abstract

**Background:**

CRIM1 is involved in the development and preservation of the nervous system, capillary development, and vascular maintenance. Although CRIM1 was reported to involve in multiple cancers, its role in breast cancer is unclear.

**Methods:**

We investigated CRIM1 expression levels using Oncomine, HPA, and immunohistochemistry analyses. BC-GenExMiner was employed to evaluate the relationship of CRIM1 expression with the clinicopathological characteristics of breast cancer. Its association with breast cancer prognosis was assessed by Kaplan-Meier analysis and PrognoScan. The correlation of the expression of CRIM1 with tumor immune infiltration was explored *via* TIMER. Gene set enrichment analysis (GSEA) was utilized to determine the cascades that are linked to CRIM1 in breast cancer. Finally, we explored CRIM1 and its co-expressed genes using R (3.6.3).

**Results:**

Here, we find that CRIM1 expression was downregulated in various subtypes of breast cancer, and it was lowest in triple-negative breast cancers. ER and PR status were positively correlated with CRIM1 expression, while HER-2 expression was negatively correlated with CRIM1 expression. But in our immunohistochemical results in breast cancer specimens collected from our laboratory, HER-2 expression was positively correlated with CRIM1 expression. The expression of CRIM1 was correlated with menopause status, T stage, pathologic stage, histological type, and P53 status but not with age, N-stage, M-stage, Radiation therapy, and BRCA1/2 status. Survival analysis found that low CRIM1 expression was correlated with poorer DMFS, RFS and OS. Notably, CRIM1 expression was positively linked to the level of infiltration by CD8^+^ T-cells, endothelial cells, and neutrophils, and negatively linked to NK, B-cells, CD4^+^ T-cells, tumor purity, macrophage M1, and Tregs. Besides, DIXDC1 and PFDN6 were correlated to CRIM1 possibly.

**Conclusion:**

Our findings demonstrated that low CRIM1 expression predict poor prognosis of breast cancer and CRIM1 might be used as a possible treatment target or prognostic marker in breast cancer. More researches are needed to better understand the prognostic value of CRIM1 in breast cancer.

## Introduction

Breast cancer is one of the commonest cancers (1) and a leading cause of cancer-linked deaths, globally (2). Early diagnosis of breast cancer is crucial for successful treatment (3, 4). Clinical and pathological features, such as the size of the tumor, tumor grade, rate of lymph node metastasis, patient age, patient morbidity (5), and various biomarkers (e.g., ER, Ki-67, PR, as well as HER-2) (6, 7) are used to predict breast cancer prognosis. However, these methods have many limitations. Thus, novel, effective prognostic biomarkers for breast cancer are urgently needed.

CRIM1 (cysteine-rich transmembrane bone morphogenetic protein regulator 1) is a Type I transmembrane protein. Past studies had shown that CRIM1 regulated the development and preservation of the nervous system and that it influenced capillary generation and vascular maintenance. CRIM1 was suggested to function as a BMP antagonist (8). CRIM1 was also reported to regulate the adhesion and migration of lung cancer cells (9). Its downregulation reduced the expression of E-cadherin, indicating that CRIM1 may inhibit EMT by suppressing cell migration and invasion (10). CRIM1 also promoted the expression of leukemia suppressor receptors by regulating miR-93 and miR-182 *via* circCRIM1 (11). Thus, CRIM1 may have therapeutic or biomarker potential against cancer.

In this study, we used bioinformatics analysis of data from several large online databases to examine gene expression differences in tumors vs normal samples, and to determine the relationship of its expression with clinicopathological features. Besides, we examined the expression of CRIM1 in breast cancer tissues by immunohistochemistry. And then, the prognostic significance of CRIM1 and its co-expressed genes in breast cancer were assessed.

## Materials and Methods

### Oncomine Analysis

Oncomine data resource (http://www.oncomine.org) is a cancer microarray repository and comprehensive data mining platform. Differential expression analysis of most cancers and their subtypes versus their normal tissues was performed (12). We used *p*=1e-4, fold change=2, and gene rank=top10% as significance thresholds for determining differences between CRIM1 levels in breast cancer vs normal tissues, as well as to identify genes that are co-expressed with CRIM1.

### Human Protein Atlas

The Human Protein Atlas is an online tool (www.proteatlas.org) on which immunohistochemistry-centered protein expression patterns of normal tissues, cancers along with cell lines are freely available (13). Currently Survival data are available for >900,000 patients (14). Imaging data on differences in CRIM1 expression in non-malignant breast tissues versus breast cancer tissues were obtained from HPA.

### Immunohistochemistry

This study involved 265 cases of invasive breast cancer patients diagnosed at the First Affiliated Hospital, Hengyang Medical School, the University of South China from 2010 to 2012. Patients underwent curative surgical treatment (mastectomy or breast-conserving surgery with axillary evaluation) and did not receive any chemotherapy or radiation therapy before surgery. We used IHC to examine CRIM1 expression levels in breast cancer tissues vs non-malignant breast tissues. Tissues were fixed using 4% formaldehyde, dehydrated in ethanol, paraffin-embedded, then sectioned continuously at 5μm thickness. The concentration of the antibody was 1:50. Antigen retrieval was done by autoclaving the sections in LBP buffer pH 6.0. They were then inoculated with H_2_O_2_ for 10 minutes for blocking the activity of endogenous peroxidase, followed by overnight inoculation with a primary antibody (1:50) at 4°C. Afterwards, we rinsed the sections with TBS and inoculated with a secondary antibody. They were next rinsed in TBS, and counter-staining using hematoxylin was done. Signal was then developed using DBA, followed by sample dehydration with ethanol, and mounting using neutral resin. They were then examined microscopically and CRIM1 expression scored as negative (-), weak (+), moderate (++), or strong (+++) staining. All statistical data analyses were done on SPSS 25.0 and data between groups were compared using the chi-square test, with *p=*<0.05 signifying statistical significance. This study was approved by the hospital ethics committee.

### Breast Cancer Gene-Expression Miner

Breast cancer gene-expression miner v4.6 (bc-GenExMiner v4.6, http://bcgenex.ico.unicancer.fr/) is a breast cancer gene expression mining program. Bc-GenExMiner is employed to examine the relationship of molecular subtypes or gene expression patterns, with disease prognosis (15–17). Here, we used bc-GenExMiner to determine the relationship of different clinical features with CRIM1 expression.

### TIMER 2.0 Database

TIMER 2.0 data resource (http://timer.cistrome.org/) is a platform for comprehensive assessment of invading immune cells in various cancers. Timer offers 6 primary analysis modules, which allow users to interactively assess the association of immune invasion with somatic copy number alterations, gene expression, clinical outcomes, as well as somatic mutations (18). Timer 2.0 uses six advanced algorithms to analyze data from more than 10,000 samples from TCGA, providing users with more reliable estimates of immune invasion levels (19, 20). Here, we analyzed CRIM1 expression in breast cancer and its correlation with the abundance of B-cells, neutrophils, CD4^+^ T-cells, NK cells, endothelial cells, CD8^+^ T-cells, macrophages, Tregs, and tumor purity.

### Kaplan-Meier Survival Analysis

The Kaplan-Meier Plotter data tool (http://kmplot.com/analysis/) is an online resource for plotting survival maps that can be utilized to explore the relationship of diverse gene expression levels of various with the clinical outcomes of individuals with breast cancer. RNA-seq, survival data, along with HTSeq counts for 26 diverse tumor types are obtained from the TCGA data resource (21, 22). Here, we assessed the association of CRIM1 expression with DMFS (distant metastasis-free survival), OS (overall survival), and RFS (relapse-free survival) in breast cancer cohorts. Hazard ratios along with log P values with 95% confidence intervals were calculated and displayed.

### PrognoScan

Prognoscan (http://www.prognoscan.org/), a large collection of cancer microarray datasets with clinical annotation, is a tool for assessing biological relationships between gene expression and prognosis. Here, we used Prognoscan to determine the relationship between CRIM1 levels and prognosis (23). Automatic calculation of COX P-values and hazard ratios (HR) with 95% confidence intervals was done based on mRNA levels.

### Gene Set Enrichment Analysis

Gene set enrichment analysis (GSEA), which is utilized in interpreting gene expression data, reveals many common biological pathways (24). Here, GSEA was done using the R package, clusterProfiler (25). Ggplot2 (3.6.3) (26) was employed to determine the biological cascades linked to CRIM1 and the rate of breast cancer in ‘CRIM1 low’ vs ‘CRIM1 high’ groups using FDR = <0.25 and adjusted *p*=<0.05 as significance thresholds.

## Results

### Expression of CRIM1 is Downregulated in Breast Cancer

**Figure 1:**
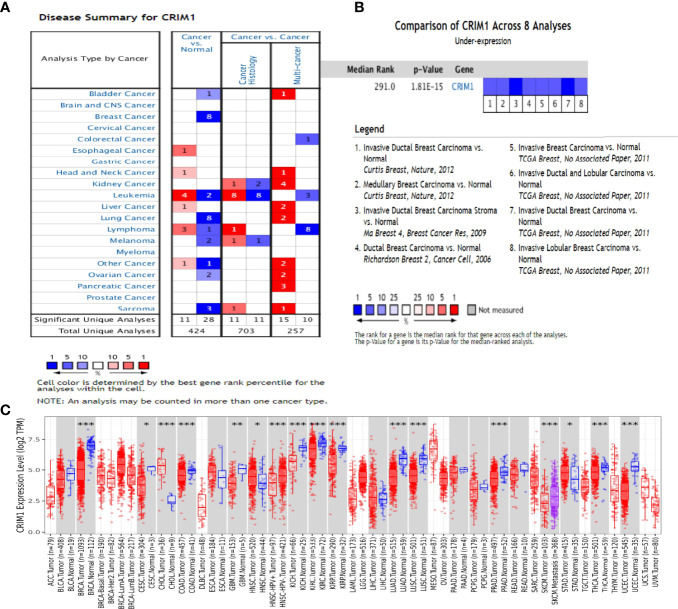
The expression of CRIM1 in various human cancers (ONCOMINE data resource). **(A)** Increased or decreased CRIM1 levels in different cancer datasets in contrast with non-malignant tissues. Red = upregulation, blue = downregulation. The lighter the color, the more meaningful it is. **(B)** Eight analyses show low levels of CRIM1 in breast cancer. **(C)** Expression of CRIM1 in distinct types of human tumors in the TIMER database. *p<0.05, **p<0.01, ***p<0.001.

Oncomine assessment exhibited that CRIM1 was remarkably downregulated in breast cancer compared with normal tissue, including in infiltrative ductal carcinoma, infiltrative lobular carcinoma, and medullary carcinoma (**Figure 1**; **Table 1**).

**Table 1:** CRIM1 expression in different subtypes of breast cancer and normal tissues using the Oncomine database.

Breast cancer subtype	P-value*	t test	Fold change	Patient number	Reference
Invasive Ductal Breast Carcinoma	3.28E-42	-21.901	-4.038	389	TCGA
Invasive Lobular Breast Carcinoma	3.63E-15	-10.071	-2.722	36	TCGA
Invasive Breast Carcinoma	8.33E-21	-11.021	-2.583	76	TCGA
Invasive Ductal and Lobular Carcinoma	9.81E-05	-6.407	-2.026	3	TCGA
Ductal Breast Carcinoma	4.41E-10	-7.754	-2.93	40	PMID: 16473279
Medullary Breast Carcinoma	4.51E-20	-16.058	-3.276	32	PMID: 22522925
Invasive Ductal Breast Carcinoma	2.48E-67	-26.654	-2.306	1556	PMID: 22522925
Invasive Ductal Breast Carcinoma	2.38E-05	-5.235	-2.312	9	PMID:19187537

*Statistical significance was determined by the Student’s t test.

**Figure 2:**
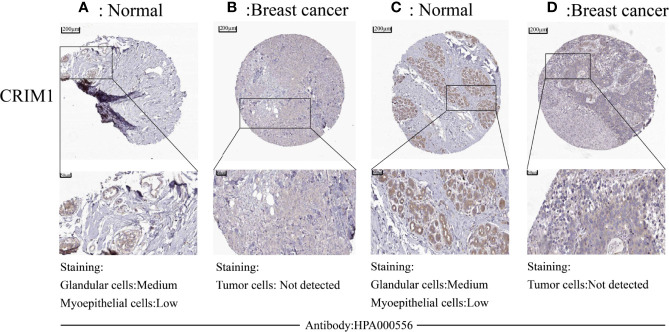
Downregulation of protein levels in breast cancer tissues was observed in human protein atlas. CRIM1 protein levels were medium in glandular cells and low in myoepithelial cells **(A, C)**, and were negative in tumor cells **(B, D)**.

Analysis of CRIM1 protein levels in breast cancer on human protein atlas exhibited that CRIM1 protein was not expressed in cancer cells (**Figures 2B, D**), although it was modestly and moderately expressed in myoepithelial cells and glandular cells, respectively (**Figures 2A, C**).

**Figure 3:**
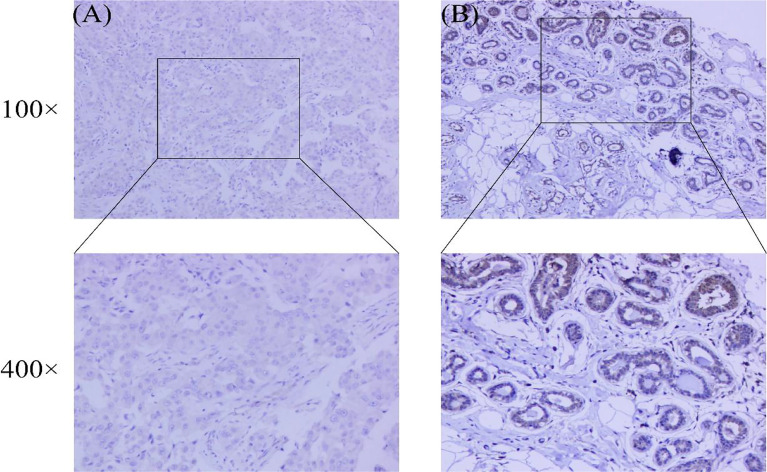
Expression of CRIM1 in 265 breast cancer patient samples and 101 normal breast tissues were examined by immunohistochemistry. **(A)** CRIM1 expression is negative (-) in breast cancer; **(B)** CRIM1 expression is moderate (++) in normal breast tissues.

In addition, we performed immunohistochemistry on breast cancer and normal tissues using anti-CRIM1 antibody(ab272542) and analyzed the results. This analysis revealed that relative to normal tissues, CRIM1 levels were remarkably lower in breast cancer tissues (**Figures 3A, B**, χ2=10.444, *p*=0.001). The rate of CRIM1 positivity in normal breast tissues was 58.42%, relative to 39.62% in carcinoma. Overall, whether the protein expression or mRNA expression of CRIM1 were lower in breast cancer than in normal breast tissues.

### Relationship Between CRIM1 Expression and Breast Cancer Clinicopathologic Features

CRIM1 expression in luminal A, luminal B, HER-2-positive, and triple-negative breast cancer tissues collected in our laboratory is shown in **Table 2**. By chi-square test, the difference regarding the expression of CRIM1 in the above four groups was statistically significant (χ2 = 16.377, P=0.001). The expression of CRIM1 in triple-negative group was lowest, but in HER-2-positive group was highest. We compared these four sets of data in pairs. It showed significant difference in CRIM1 expression between the HER-2-positive group with the Luminal A, Luminal B, and triple-negative groups (χ2 = 7.402, p=0.007; χ2 = 13.516, p=0.000; χ2 = 13.650, p=0.000). However, there was no significant difference between Luminal A group with Luminal B and TNBC groups (χ2 = 0.408, p=0.523; χ2 = 1.334, p=0.248), nor between Luminal B and TNBC groups (χ2 = 0.579, p=0.447).

**Table 2:** CRIM1 expression in different subtypes of breast cancer and normal tissues using Immunohistochemistry [n(%)].

clinicopathological features	number	CRIM1 expression		χ2	*P*-value
		negative	positive		
group					
breast cancer tissues	265	160 (60.4)	105 (39.6)		
normal breast tissues	101	42 (41.6)	59 (58.4)	10.444	0.001
pathological type					
Luminal A	36	22 (61.1)	14 (38.9)		
Luminal B	103	69 (67.0)	34 (33.0)		
HER2	21	5 (23.8)	16 (76.2)		
TNBC	38	38 (73.7)	10 (26.3)	16.377	0.001

**Figure 4:**
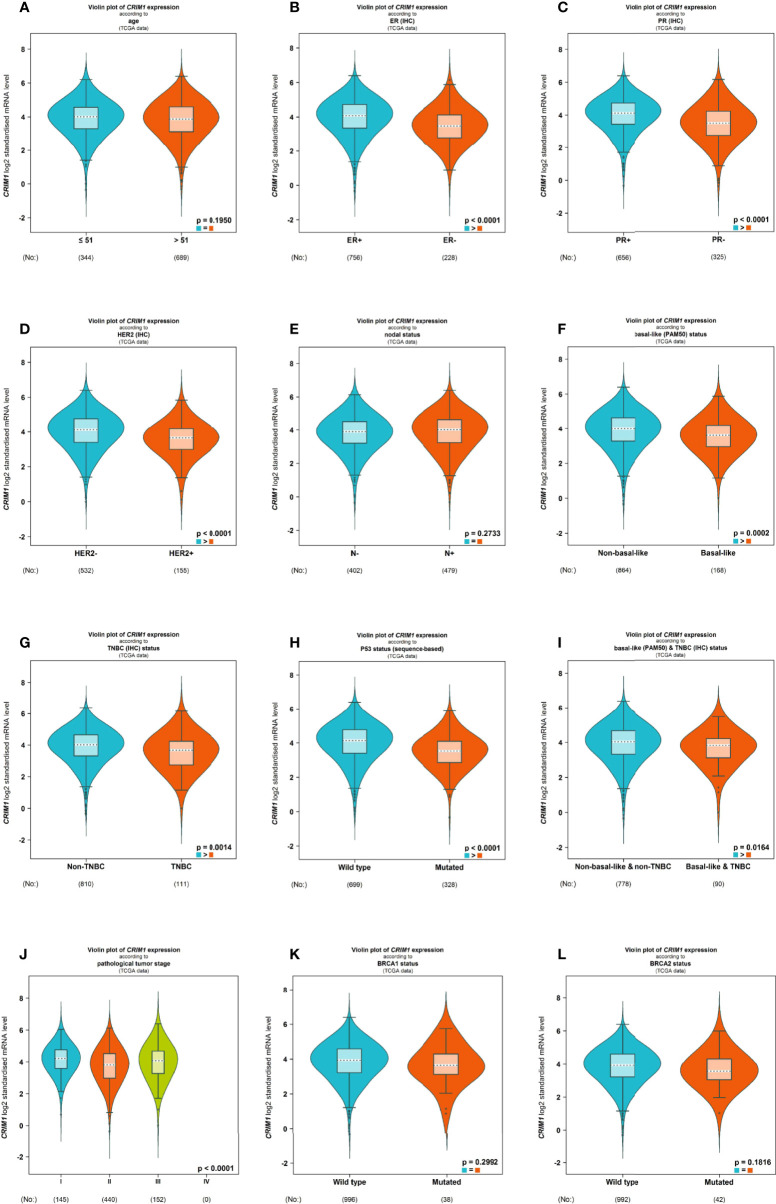
Violin plot illustrated the relationship of CRIM1 expression with clinical indicators using bc-GenExMiner. Data include age **(A)**, ER **(B)**, PR **(C)**, HER-2 **(D)**, nodal status **(E)**, basal-like status **(F)**, TNBC status **(G)**, P53 status **(H)**, basal-like & TNBC status **(I)**, pathological tumor stage **(J)**, BRCA1 status **(K)** and BRCA2 status **(L)**.

Next, we divided breast cancer patients into various groups according to various clinical characteristics and compared differences in CRIM1 mRNA levels using bc-GenExMiner. The groups did not differ remarkably with regards to age and nodal status (**Figures 4A, E**). ER and PR status were positively correlated with CRIM1 expression (**Figures 4B, C**), while HER-2 expression was negatively correlated with CRIM1 expression (**Figure 4D**). That was somewhat different from our immunohistochemical results in breast cancer specimens collected in our laboratory. We also found that CRIM1 was remarkably downregulated in the basal-like subtype when compared to the non-basal-like subtype. Similar observations were made for triple-negative breast cancer (TNBC, **Figures 4F, G, I**). P53 is one of the most commonly mutated oncogenes (27). Our analysis found that CRIM1 was remarkably downregulated in the p53 mutant group than the p53 wild-type group (**Figure 4H**). We further found that the expression of CRIM1 had a correlation with the pathological tumor stage of breast cancer, and it was obvious to observe that its expression was highest in stage I (**Figure 4J**). And there was no relevance between CRIM1 expression and BRCA1/2 status (**Figures 4K, L**). Next, we evaluated CRIM1 expression in clinical specimens in relation to breast cancer clinicopathological parameters. Breast cancer patients were then grouped into the high- and low-CRIM1 expression groups and their correlation with age, menopause, TNM stage, histological type, pathologic stage, HER2 status, and radiotherapy were investigated. CRIM1 expression correlated with menopause status(p=0.005), T stage(p=0.002), pathologic stage(p=0.040), histological type(p=0.011), and HER2 status(p<0.001) but not with age(p=0.411), N-stage(p=0.372), M-stage(p=0.219), and Radiation therapy (p=0.435) (**Table 3**).

**Table 3:** Relationship between CRIM1 expression and clinicopathologic parameters of breast cancer patients.

Characteristic	Low expression of CRIM1	High expression of CRIM1	p
n	541	542	
Age, n (%)			0.411
<=60	293 (27.1%)	308 (28.4%)	
>60	248 (22.9%)	234 (21.6%)	
Menopause status, n (%)			**0.005**
Pre	92 (9.5%)	137 (14.1%)	
Peri	20 (2.1%)	20 (2.1%)	
Post	370 (38.1%)	333 (34.3%)	
T stage, n (%)			**0.002**
T1	112 (10.4%)	165 (15.3%)	
T2	338 (31.3%)	291 (26.9%)	
T3	69 (6.4%)	70 (6.5%)	
T4	20 (1.9%)	15 (1.4%)	
N stage, n (%)			0.372
N0	255 (24%)	259 (24.3%)	
N1	182 (17.1%)	176 (16.5%)	
N2	51 (4.8%)	65 (6.1%)	
N3	43 (4%)	33 (3.1%)	
M stage, n (%)			0.219
M0	438 (47.5%)	464 (50.3%)	
M1	13 (1.4%)	7 (0.8%)	
Pathologic stage, n (%)			**0.040**
Stage I	74 (7%)	107 (10.1%)	
Stage II	324 (30.6%)	295 (27.8%)	
Stage III	123 (11.6%)	119 (11.2%)	
Stage IV	11 (1%)	7 (0.7%)	
Histological type, n (%)			**0.011**
Infiltrating Ductal Carcinoma	407 (41.7%)	365 (37.4%)	
Infiltrating Lobular Carcinoma	87 (8.9%)	118 (12.1%)	
HER2 status, n (%)			**< 0.001**
Negative	239 (32.9%)	319 (43.9%)	
Indeterminate	7 (1%)	5 (0.7%)	
Positive	99 (13.6%)	58 (8%)	
Radiation therapy, n (%)			0.435
No	226 (22.9%)	208 (21.1%)	
Yes	273 (27.7%)	280 (28.4%)	

Values in bold indicate that the results are statistically significant.

### Decreased CRIM1 Expression Impacted the Prognosis of Breast Cancer Patients

**Figure 5:**
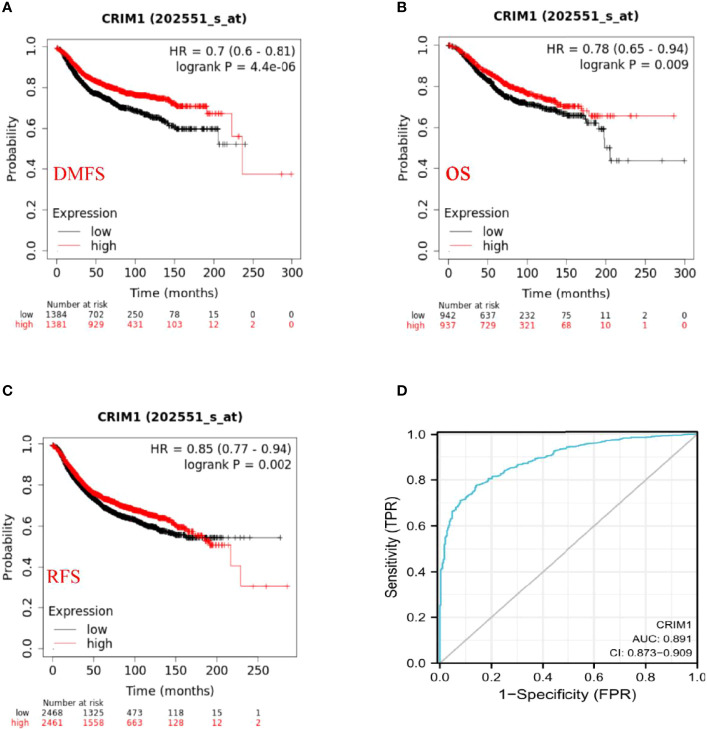
Prognostic significance of CRIM1 expression in breast cancer **(A–D)**. **(A–C)** Kaplan-Meier analysis revealed that high CRIM1 levels were correlated with better DMFS (distant metastasis-free survival), OS (overall survival), and RFS (relapse-free survival). **(D)** Receiver operating characteristic (ROC) curve analysis of the correlation between CRIM1 and survival in breast cancer patients showed that CRIM1 had good performance in predicting breast cancer survival.

Kaplan-Meier and log-rank test analyses of the significance of CRIM1 expression on breast prognosis revealed that higher CRIM1 levels correlated with better DMFS (HR=0.7, *p*=4.4e-06), OS (HR=0.78, *p*=0.009), and RFS (HR=0.85, *p*=0.002). However, low CRIM1 levels were correlated with worse survival (**Figures 5A–C**). Similar observations were made using PrognoScan analysis which exhibited that low CRIM1 expression remarkably linked to poorer RFS, OS, DSS, DFS, and DMFS (**Table 4**). Receiver operating characteristic (ROC) curve analysis of the relationship of CRIM1 with survival time of breast cancer patients revealed an AUC of 0.891 (**Figure 5D**), indicating good performance for CRIM1 in predicting survival.

**Table 4:** CRIM1 expression and survival data of breast cancer patients through the PrognoScan database.

DATASET	ENDPOINT	PROBE ID	N	COX P-VALUE	HR [95% CI-low- CI-up]
GSE1456-GPL96	Relapse Free Survival	202552_s_at	159	2.71452E-05	0.35 [0.21 - 0.57]
GSE1456-GPL96	Disease Specific Survival	202552_s_at	159	0.000165802	0.32 [0.18 - 0.58]
GSE1456-GPL96	Overall Survival	202552_s_at	159	0.0277991	0.55 [0.33 - 0.94]
GSE1456-GPL97	Overall Survival	228496_s_at	159	0.0436275	0.64 [0.41 - 0.99]
GSE1456-GPL97	Relapse Free Survival	228496_s_at	159	0.000131085	0.47 [0.32 - 0.69]
GSE1456-GPL97	Disease Specific Survival	228496_s_at	159	9.81953E-05	0.42 [0.27 - 0.65]
GSE7378	Disease Free Survival	202552_s_at	54	0.0470036	0.47 [0.23 - 0.99]
GSE3494-GPL96	Disease Specific Survival	202552_s_at	236	0.00154078	0.39 [0.22 - 0.70]
GSE3494-GPL97	Disease Specific Survival	228496_s_at	236	0.0100557	0.56 [0.36 - 0.87]
GSE4922-GPL96	Disease Free Survival	202552_s_at	249	0.00532698	0.55 [0.36 - 0.84]
GSE4922-GPL97	Disease Free Survival	228496_s_at	249	0.00296997	0.62 [0.45 - 0.85]
GSE2990	Relapse Free Survival	202552_s_at	62	0.0181867	0.49 [0.27 - 0.88]
GSE2990	Distant Metastasis Free Survival	202552_s_at	54	0.0170378	0.38 [0.17 - 0.84]

### CRIM1 Expression Levels Correlated With Immune Cells Invasion Levels in Breast Cancer

**Figure 6:**
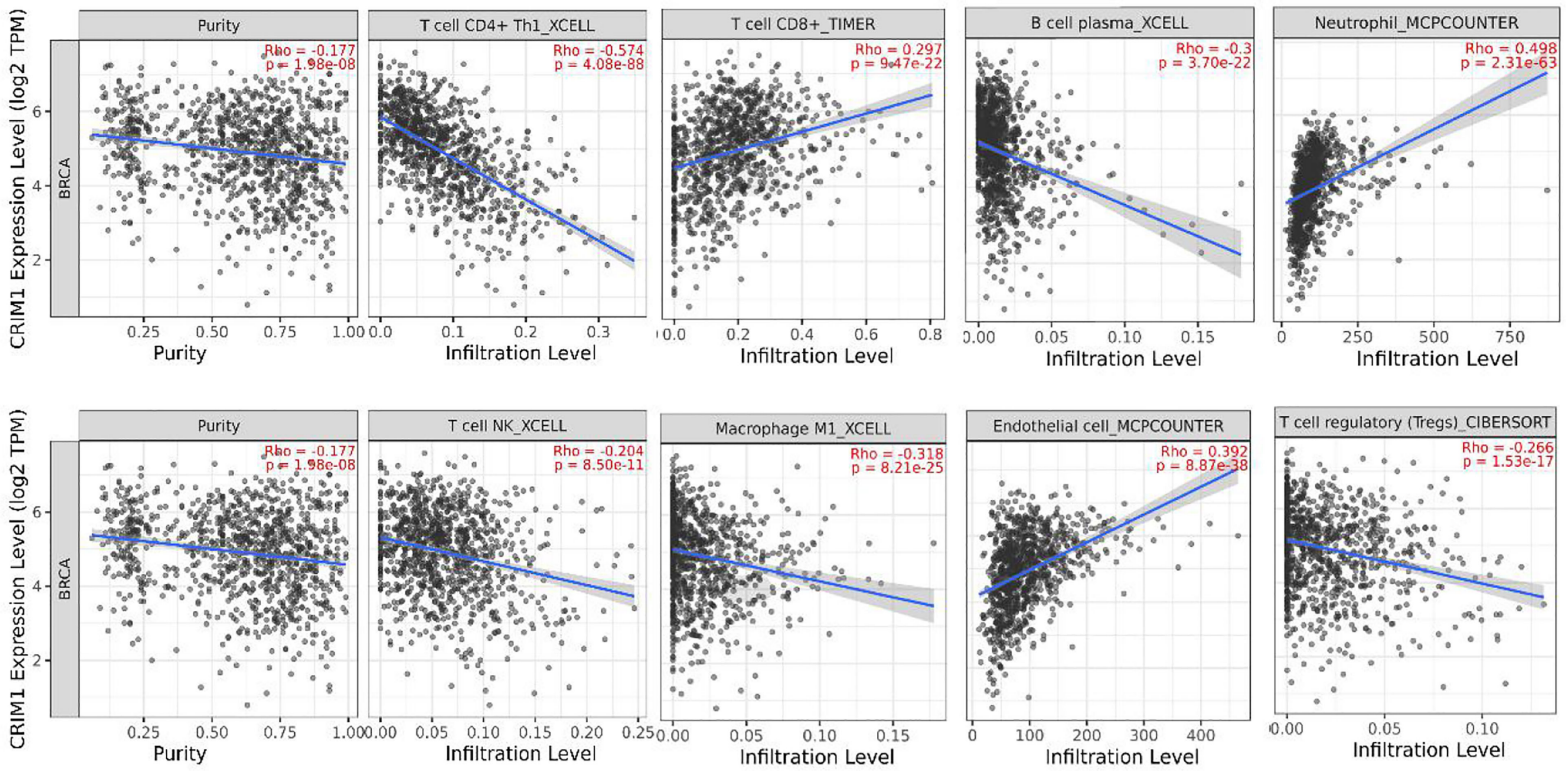
TIMER analysis of the relationship of CRIM1 expression with immune cells invasion levels in breast cancer. CRIM1 expression in breast cancer tissues were negatively correlated with tumor purity, Tregs, NK, B-cells, and CD4^+^ T-cells, macrophage M1, and positively correlated with CD8^+^ T-cells, endothelial cells, and neutrophils (n=1100).

Further, we adopted TIMER to examine the relationship of CRIM1 with invading immune cells in breast cancer, consisting of CD4^+^ T-cells, neutrophils, CD8^+^ T-cells, macrophage M1, B-cells, NK, endothelial cells, and Tregs. This analysis illustrated that CRIM1 expression was positively linked to CD8^+^ T-cells (r=0.297, *p*=9.47e-22), endothelial cells (r=0.392, *p*=8.87e-38) and neutrophils (r=0.498, *p*=2.31e-63), and negatively linked to NK (r=-0.204, *p*=8.50e-11), B-cells (r=-0.3, *p*=3.70e-22), CD4^+^ T-cells (r=-0.574, *p*=4.08e-88), tumor purity (r=-0.177, *p*=1.98e-08), macrophage M1(r=-0.318, p=8.21e-25), and Tregs (r=-0.266, *p*=1.53e-17) (**Figure 6**). These findings indicated that CRIM1 levels may regulate immune cells infiltration in breast cancer.

### GSEA Identified CRIM1-Associated Pathways in Breast Cancer

**Figure 7:**
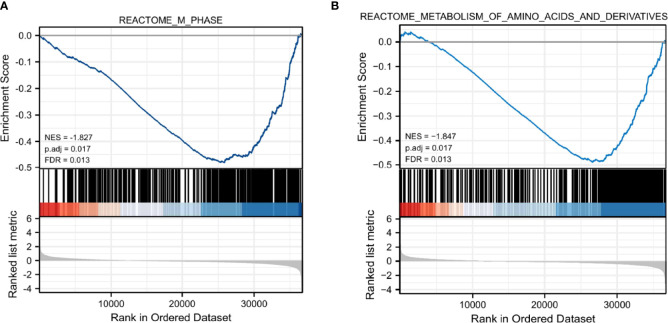
GSEA revealed that M phase **(A)** and metabolism of amino acids and derivatives **(B)** were enriched in the low CRIM1 expression group. NES, normalized ES; FDR, false discovery rate.

To evaluate the possible mechanisms accounting for the effects of CRIM1 on breast cancer, we performed GSEA analysis on samples with low CRIM1 levels relative to those with high CRIM1 levels. This analysis revealed significant differences in the abundance of MSigDB collections (c2.cp.v7.2.symbols.gmt) and that M phase, metabolism of amino acids and derivatives were remarkably differentially enriched in samples with low CRIM1 levels (**Figures 7A, B**), indicating that low CRIM1 levels may influence breast cancer development *via* these pathways.

### CRIM1, PFDN6, and DIXDC1 Were Co-Expressed in Breast Cancer Patients

**Figure 8:**
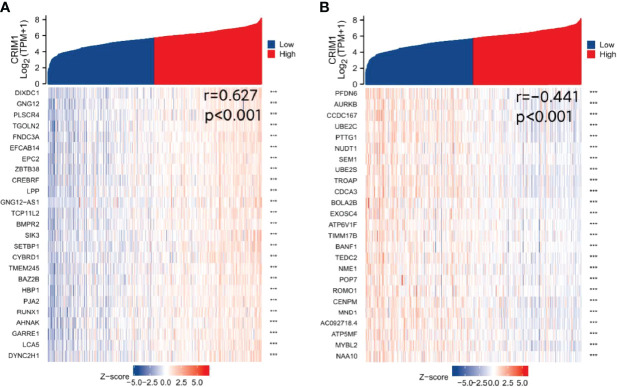
Identification of genes that are co-expressed with CRIM1. **(A)** DIXDC1 was positively correlated with CRIM1. **(B)** PFDN6 was negatively correlated with CRIM1. ***p=<0.001.

Finally, we analyzed TCGA data resources (https://portal.gdc.cancer.gov/) BRCA RNAseq (data in level 3 HTSeq-FPKM format) for genes that are co-expressed with CRIM1 using the R packages, stat (28) and ggplot2 (26) (3.6.3). This analysis identified 5254 IDs that met the cutoff threshold of |cor|>0.3, *p*=<0.05. Results showed that 5091 genes were positively correlated with CRIM1 levels, while 163 genes were negatively correlated with CRIM1. Notably, DIXDC1 had a strong positive correlation with CRIM1, while PFDN6 had a strong negative association with CRIM1 (**Figures 8A, B**). It was reported that DIXDC1 deletion enhances SNAIL-dependent gene expression, which enhances invasion and remodeling of the tumor microenvironment (29). PFDN6 (Prefoldin 6) is a β-like subunit of hetero-hexameric chaperone proteins (30). Prefoldins are highly specialized co-chaperones of actin and microtubule protein folding (31).

## Discussion

Despite advances in chemotherapy, radiotherapy, and surgical treatment of breast cancer, its survival rate remains low (32). Breast cancer is the primary cause of mortality along with morbidity in women (33). Thus, new breast cancer biomarkers are urgently needed for better outcomes.

CRIM1 is involved in histogenesis *via* its interaction with growth factors, including bone morphogenetic protein (BMP), vascular endothelial growth factor (VEGF), as well as transforming growth factor-beta (TGF-β) (34). Reduced CRIM1 expression is associated with reduced E-cadherin levels. CRIM1 suppresses cell migration and invasion by regulating the expression of various EMT-related factors (10). However, the significance of CRIM1 expression in breast cancer is unclear.

Oncomine analysis of CRIM1 expression in breast cancer revealed decreased CRIM1 levels in many kinds of breast carcinoma, such as infiltrative ductal breast carcinoma, infiltrative lobular breast carcinoma, and medullary breast carcinoma. Analysis using bc-GenExMiner revealed that ER and PR status were positively associated with CRIM1 expression, while HER-2, basal-like status, triple-negative status along with p53 status were negatively correlated with CRIM1 expression in breast cancer tissues. That was somewhat different from our immunohistochemical results in breast cancer specimens collected in our laboratory. In our immunohistochemical results, HER-2 expression was positively correlated with CRIM1 expression. More experimental evidence is needed to verify. Human Protein Atlas results showed that CRIM1 expression was lower than normal in breast cancer and IHC analyses revealed the expression of CRIM1 was lowest in triple-negative breast cancer. The pathological tumor stage of breast cancer was in connection with CRIM1 expression, with the highest expression in stage I. Expression of CRIM1 was correlated with menopause status, T stage, pathologic stage, histological type, and P53 status but not with age, N-stage, M-stage, and Radiation therapy.

Investigation of the impact of CRIM1 on breast cancer prognosis using Kaplan-Meier and PrognoScan analyses revealed that reduced CRIM1 levels correlated with poorer RFS, OS, DSS, DFS, and DMFS. These findings indicated that low CRIM1 expression may effectively predict breast cancer prognosis.

TIMER analysis of the mechanisms underlying CRIM1 effects on breast cancer revealed that CRIM1 levels were negatively linked to tumor purity, macrophage M1, Tregs, NK cells, B-cells, and CD4^+^ T-cells, but were positively correlated with CD8^+^ T-cells, endothelial cells, macrophage M1 as well as neutrophils.

Using co-expression along with correlation analyses, we found that DIXDC1 was positively correlated with CRIM1 expression, while PFDN6 expression was negatively related to CRIM1 levels. DIXDC1 is a scaffolding protein consisting of 3 protein-interacting structural domains (an actin-binding calponin homology (CH) domain, a coiled-coil (CC) domain, and a Dishevelled-Axin (DIX) oligomerization domain). Numerous studies have reported its positive regulation of the DIX structural domain in Wnt signaling. Reduced DIXDC1 expression promotes malignant behavior and is associated with the survival in cancer (29, 35).

In conclusion, our findings indicated that CRIM1 was downregulated in breast cancer in contrast with non-malignant tissues and that reduced CRIM1 expression was correlated with poor prognosis. Our studies were mainly based on bioinformatics analysis and lacked systematic experimental validation. Further researches are needed to better characterize the prognostic value of CRIM1 in breast cancer.

## Data Availability Statement

Publicly available datasets were analyzed in this study. This data can be found here: TCGA (https://portal.gdc.cancer.gov/) RNAseq data in level 3 HTSeq - Counts format from the BRCA (Breast Invasive Cancer) project.

## Ethics Statement

This study was approved by the Ethics Committee of the First Affiliated Hospital of University of South China (Approval number:2020ll1010014). The patients/participants provided their written informed consent to participate in this study.

## Author Contributions

RH and YL participated in the designing of experiments. Experiments were conducted with WW, BJ, XC, LX, and XZ participated in analyzing and interpreting data. WW, YL and RH were the main writers of the manuscript. All authors contributed to the article and approved the submitted version.

## Funding

This work was funded by the Natural Science Foundation of Hunan Province (2019JJ80036) and the funds from the Health Committee of Hunan Province (20201974, B20180052).

## Conflict of Interest

The authors declare that the research was conducted in the absence of any commercial or financial relationships that could be construed as a potential conflict of interest.

## Publisher’s Note

All claims expressed in this article are solely those of the authors and do not necessarily represent those of their affiliated organizations, or those of the publisher, the editors and the reviewers. Any product that may be evaluated in this article, or claim that may be made by its manufacturer, is not guaranteed or endorsed by the publisher.

## Abbreviations

CRIM1, cysteine-rich transmembrane bone morphogenetic protein regulator 1; BC, breast cancer; DIX, Dishevelled-Axin; GSEA, Gene set enrichment analysis; ER, estrogen receptor; PR, progesterone receptor; HER-2, human epidermal growth factor receptor 2; DMFS, distant metastasis-free surviva l; RFS, relapse-free survival; OS, overall survival; IHC, Immunohistochemistry; BMP, bone morphogenetic protein; VEGF, vascular endothelial growth factor; TGF-b, transforming growth factor-beta.
